# Analysis of four *achaete-scute *homologs in *Bombyx mori *reveals new viewpoints of the evolution and functions of this gene family

**DOI:** 10.1186/1471-2156-9-24

**Published:** 2008-03-06

**Authors:** Qingxiang Zhou, Tianyi Zhang, Weihua Xu, Linlin Yu, Yongzhu Yi, Zhifang Zhang

**Affiliations:** 1 The Biotechnology Research Institute, National Engineering of crop germplasm and genetic improvement, Chinese Academy of Agricultural Sciences, Beijing, 100081, China; 2 State Key Laboratory for Biocontrol and Institute of Entomology, School of Life Sciences, SUN YAT-SEN University, Guangzhou 510275, China

## Abstract

**Background:**

*achaete-scute *complexe (*AS-C*) has been widely studied at genetic, developmental and evolutional levels. Genes of this family encode proteins containing a highly conserved bHLH domain, which take part in the regulation of the development of central nervous system and peripheral nervous system. Many *AS-C *homologs have been isolated from various vertebrates and invertebrates. Also, *AS-C *genes are duplicated during the evolution of Diptera. Functions besides neural development controlling have also been found in *Drosophila AS-C *genes.

**Results:**

We cloned four *achaete-scute *homologs (*ASH*) from the lepidopteran model organism *Bombyx mori*, including three proneural genes and one neural precursor gene. Proteins encoded by them contained the characteristic bHLH domain and the three proneural ones were also found to have the C-terminal conserved motif. These genes regulated promoter activity through the Class A E-boxes *in vitro*. Though both *Bm-ASH *and *Drosophila AS-C *have four members, they are not in one by one corresponding relationships. Results of RT-PCR and real-time PCR showed that *Bm-ASH *genes were expressed in different larval tissues, and had well-regulated expressional profiles during the development of embryo and wing/wing disc.

**Conclusion:**

There are four *achaete-scute *homologs in *Bombyx mori*, the second insect having four *AS-C *genes so far, and these genes have multiple functions in silkworm life cycle. *AS-C *gene duplication in insects occurs after or parallel to, but not before the taxonomic order formation during evolution.

## Background

Transcriptional factors of the bHLH (basic helix-loop-helix) family play important roles in the development of metazoan, taking part in the regulation of neurogenesis, myogenesis, haemopoiesis and so on [1-5]. The *achaete-scute *complex (*AS-C*), a group of four bHLH genes, has been found to be involved in the determination of *Drosophila *neural precursors [6-9].

AS-C proteins interact with another bHLH protein Daughterless (Da) to form a dimer, and bind Class A E-boxes CAGG/CTG [10]. AS-C/Da heterodimers' binding sites were found in the upstream regions of *hunchback *(*hb*) [10] and *phyllopod *(*phyl*) [11]. In *Drosophila*, they were also found in the upstream region of *ac *itself, and required for auto- and cross-regulation by *ac *and *sc *[12].

The main functions of *AS-C *genes are regulating the formation and patterning of precursors of central nervous system (CNS) and peripheral nervous system (PNS). During neural development, *AS-C *genes are expressed in one or more cells within the proneural clusters, which are committed to the neural precursor fate, and the remaining cells in the cluster differentiate to be epidermal cells or are eliminated by apoptosis [8,13,14]. *Tribolium castaneum ASH *(*Tc-ASH*) and *Hydra vulgaris cnidarian ASH *(*Cn-ASH*) show similar functions during the formation of sensory organs in the transgenic *Drosophila *[15,16]. Also, it was found that Cn-ASH protein formed heterodimers with *Drosophila *Da (Dm-Da) protein *in vitro*, and these dimers specially bound to the consensus E-boxes [15]. Besides regulation of neurogenesis, *AS-C *genes have other functions. *ac *takes part in the development of Malpighian tubule by expressing in the tip cell and regulating the sequential fates of the organ [17]. *sc *regulates sex determination by its different expression dosage between females and males, but neither *ac *nor *l'sc *can substitute for *sc *in this function [18-20]. *l'sc *expression is necessary for muscle founder cells segregating from the somatic mesoderm. Loss of expression of *l'sc *leads to a loss, whereas over expression causes a duplication of muscles and founder cells [21].

According to the sequence and functional analysis in different species, *AS-C *genes are conserved during evolution. The four genes of *Drosophila AS-C *are *achaete *(*ac*), *scute *(*sc*), *lethal of scute *(*l'sc*) and *asense *(*ase*). The former three are defined as proneural genes and the last one as neural precursor gene, basing on their expression patterns and functions in proneural clusters. *AS-*C homologs have been isolated from various invertebrates and vertebrates [15,16,22-34]. They all have a conserved bHLH domain and some of them also have a conserved C-terminal motif. Gene duplication events caused a dramatic increase of *AS-C *gene number during Arthropoda evolution [16,30]. Within Insecta, *AS-C *homologs have been studied in Diptera (flies and mosquitoes), Hymenoptera (bees), Coleoptera (the red flour beetle) and Lepidoptera (the butterfly *P. coenia*). Each of the insects has one *ase*-like gene. Most of them have only one proneural gene, while the medfly *Ceratitis capitata *has two, and the fruit fly has three. The four genes in *Drosophila *were believed to have arisen from a single ancestral gene by three independent duplication events [16,30]. *AS-C *homologs in the insects studied are closely linked in the chromosome, for example, the four *AS-C *genes comprising about 100 Kb in *Drosophila*, and the two residing about 22 Kb apart in *Anopheles*, about 55 Kb apart in *Tribolium*, and about 40 Kb apart in *Apis *[16,34].

The silkworm, *Bombyx mori*, is a model organism of Lepidoptera. With the completion of silkworm genomic sequencing project [35], *Bombyx mori *is emerging as an important model lepidopteran. Only one *AS-C *homolog had been isolated from lepidopteran in previous studies [26]. In the present study, we found there were four *AS-C *homologs in silkworm, three proneural genes and an *ase*-like one. We detected the transcriptional activity of the genes by transient expression in Bm-N cells. Expression profiles of the genes in different tissues and expression changes during the development course of silkworm embryo and wing disc/wing were also studied.

## Results

### Isolation and identification of *achaete-scute *homologs in *Bombyx mori*

It is well known that each *achaete-scute *homolog has a highly conserved bHLH domain which distinguishes them from other bHLH proteins. Using the amino acid sequence of *Drosophila *Ac (Genbank: AAF45498) to blast the silkworm EST database (see Materials and Methods section), we obtained an EST sequence (GenBank: CK537057) encoding a conserved *AS-C *bHLH domain. Primers were designed based on the EST sequence, and RACE assay was carried out using the midgut total RNA of 3d 5^th ^instar larva as the template. After sequencing and assembling, we gained a cDNA of 1,332 bp and named it *Bm-ASH1 *(Genbank: DQ350889). *Bm-ASH1 *gene contains a 582 bp ORF region (including the stop codon) and encodes a 193 aa protein.

Then we screened the *Bombyx mori *genome database using the 193 aa Bm-ASH1 protein sequence, and found genes with conserved *AS-C *bHLH region in four more contigs (Genbank: AADK01030307, AADK01036667, AADK01011379 and BAAB01105243), besides the two (Genbank: AADK01005140 and BAAB01089921) corresponding to *Bm-ASH1*. BAAB01105243 is part of AADK01011379. Each of them contained a deduced ORF region, and then we cloned the ORF regions by RT-PCR methods and they were sequenced. Primers for 3'-RACE were designed basing on the sequences of the ORF regions, and 3'-RACE assay was processed using the total RNA from 1 d pupal wing as the template. The segments gained by 3'-RACE were sequenced and assembled with each corresponding ORF sequence. The final cDNA sequences were 1,449 bp, 990 bp and 1,695 bp long, and were named *Bm-ASH2 *(Genbank: EF620927), *Bm-ASH3 *(Genbank: EF620928) and *Bm-ase *(Genbank: EF620929) respectively. *Bm-ASH2 *gene contained a 720 bp OFR region (including the stop codon) and encoded a 239 aa protein, *Bm-ASH3 *gene contained a 726 bp OFR region (including the stop codon) and encoded a 241 aa protein, and *Bm-ase *gene contained a 1,215 bp OFR region (including the stop codon) and encoded a 404 aa protein.

**Figure 1:**
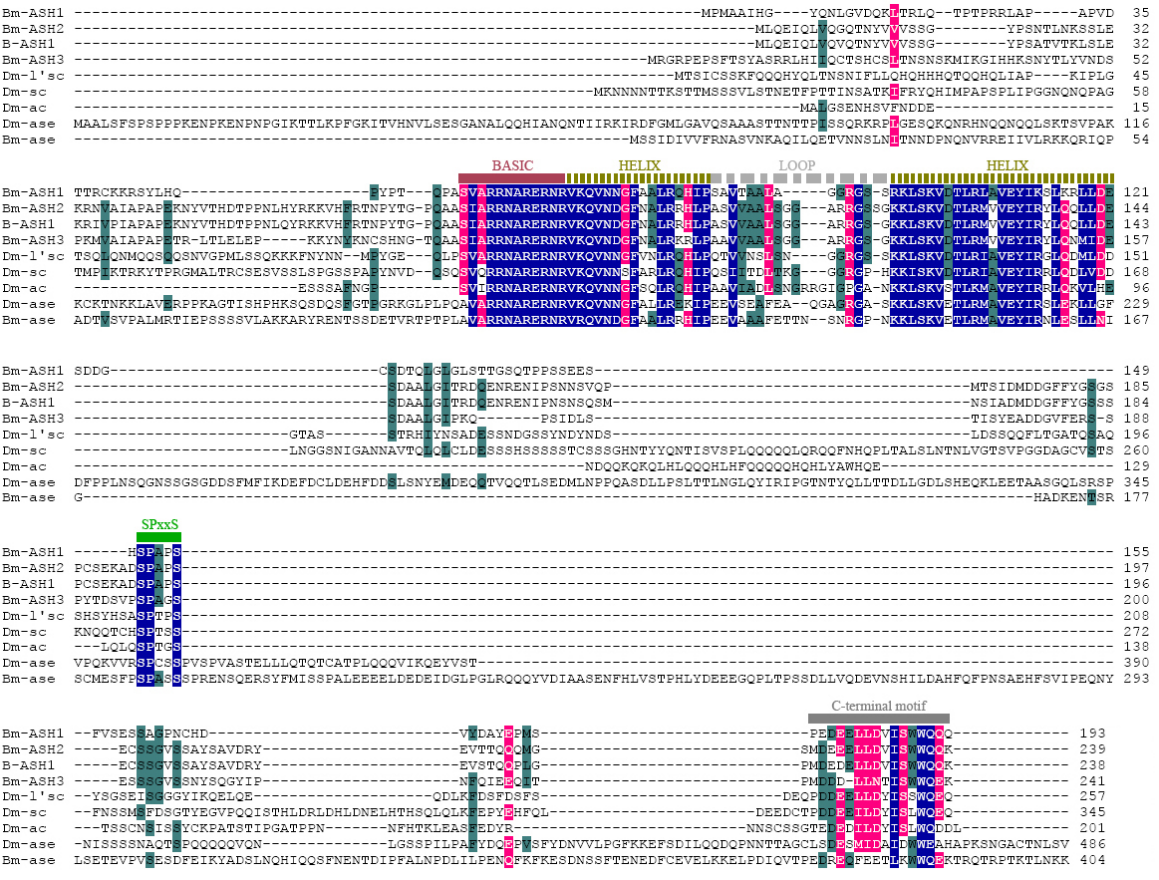
**Alignment of the amino acid sequences of proteins encoding by *Bm-ASH, B-ASH1 *and *Dm AS-C *genes**. All of the proteins contain the *AS-C *characteristic one basic and two helix domains flanking a non-conserved loop domain, and a conserved C-terminal 16–17 aa motif also exists in each of the proneural genes but neither of the *asense *genes. Moreover, a conserved short motif SPxxS is also found in all of the investigated proteins. Amino acids that are similar in 100% of aligned sequences are shaded blue, in more than 80% but less than 100% with pink, and in more than 60% but less than 80% with darkgreen.

Alignment results with *Drosophila *AS-C proteins showed that all four proteins encoded by *Bm-ASH *genes contained the *AS-C *characteristic one basic and two helix domains flanking a non-conserved loop domain (Fig. 1). Bm-ASH1, Bm-ASH2 and Bm-ASH3 also had the C-terminal 16–17 aa conserved motif, but Bm-Ase did not. There was a highly conserved short motif (SPxxS, x means uncertain acid amino) around the region of PEST domain [36-38]. In the bHLH region, Bm-ASH1 shares 74.6%, Bm-ASH2 shares 72.1% and Bm-ASH3 shares 72.1% amino acid identity with Dm-L'sc, and Bm-Ase shares 74.3% with Dm-Ase (Table 1). The data above indicate that *Bm-ASH1*, *Bm-ASH2 *and *Bm-ASH3 *are proneural genes and *Bm-ase *an *asense *homolog.

**Table 1:** Homology comparison of proteins encoded by the Bm-ASH genes with other insect ASH proteins.

**ASH name**	**Percent Identity (%)**			
	
	**Bm-ASH1**	**Bm-ASH2**	**Bm-ASH3**	**Bm-Ase**
**Bm-ASH1**	/			
**Bm-ASH2**	71.6 (44.6)	/		
**Bm-ASH3**	70.1 (36.8)	91.3 (49.8)	/	
**B-ASH1**	73.1 (38.9)	100.0 (90.3)	91.3 (47.9)	66.7 (26.5)
**Ag-ASH**	79.1 (47.7)	70.6 (34.3)	70.6 (30.7)	67.6 (19.7)
**Am-ASH**	77.6 (50.8)	67.1 (32.2)	63.8 (26.1)	68.6 (25.8)
**Dm-Ac**	68.7 (31.6)	65.7 (28.9)	65.2 (30.3)	60.0 (28.4)
**Dm-Sc**	68.7 (42.0)	62.3 (27.2)	58.0 (27.4)	59.4 (21.2)
**Dm-L'sc**	74.6 (40.9)	72.1 (31.0)	72.1 (36.9)	61.8 (27.2)
**Tc-ASH**	76.1 (46.6)	62.9 (34.3)	63.8 (26.1)	67.1 (28.1)
**Bm-Ase**	67.2 (31.1)	62.9 (30.1)	60.9 (27.0)	/
**Ag-Ase**	68.7 (30.1)	54.3 (20.9)	56.5 (21.6)	71.4 (22.0)
**Am-Ase**	73.1 (38.9)	68.8 (26.8)	62.5 (23.7)	71.9 (24.9)
**Dm-Ase**	70.1 (37.8)	57.1 (23.0)	60.9 (24.5)	74.3 (24.0)
**Tc-Ase**	70.1 (36.8)	63.2 (25.1)	58.8 (26.1)	77.9 (33.5)

The number in the bracket is the identity percent between two whole protein sequences, and the number before the bracket is the identity percent within the bHLH regions.

**Figure 2:**
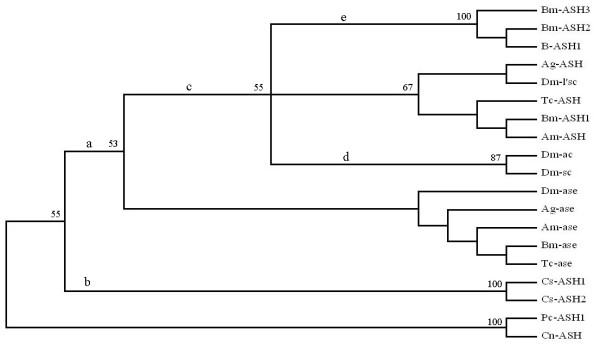
**Phylogenetic status of silkworm ASH proteins in insect**. The support values are given at each node (100 replications). Branch lengths are not drawn to scale. Letters upside of the branch lines show the duplication events as clarified by Wheeler et al. [16] (see discussion for detailed description). It is obvious that all of the Asense proteins are grouped to one clade and the proneural genes are grouped to another within the insect group. Bm-ASH2 and Bm-ASH3 are grouped to a sub-clade parallel the one which Bm-ASH1 is sorted in. Ag-ASH, *Anopheles gambiae *Achaete-Scute homolog (Genbank: AAK97461); Am-ASH,*Apis mellifera *Achaete-Scute homolog (Genbank: XP_393665); B-ASH1, Butterfly (Genbank: *Precis coenia*) Achaete-Scute homolog 1 (Genbank: AAC24714); Bm-ASH1, *Bombyx mori *Achaete-Scute homolog 1 (Genbank: NP_001037416); Bm-ASH2, *Bombyx mori *Achaete-Scute homolog 2 (Genbank: EF620927); Bm-ASH3, *Bombyx mori *Achaete-Scute homolog 3 (Genbank: EF620928); Cn-ASH, *Hydra vulgaris cnidarian *Achaete-Scute homolog (Genbank: U36275); Cs-ASH1, *Cupiennius salei *Achaete-Scute homolog 1 (Genbank: CAC27516); Cs-ASH2, *Cupiennius salei *Achaete-Scute homolog 2 (Genbank: CAC27517); Dm-ac, *Drosophila melanogaster *Achaete (Genbank: AAF45498); Dm-sc, *Drosophila melanogaster *Scute (Genbank: AAF45499); Dm-l'sc, *Drosophila melanogaster *Lethal of scute (Genbank: AAF45500); Pc-ASH1, *Podocoryne carnea *Achaete-Scute homolog 1 (Genbank: AAN85110); Tc-ASH, *Tribolium castaneum *Achaete-Scute homolog (Genbank: AAQ23386); Ag-ase, *Anopheles gambiae *Asense (Genbank: AAAB01008963); Am-ase, *Apis mellifera *Asense (Genbank: XP_393664); Bm-ase, *Bombyx mori *Asense (Genbank: EF620929); Dm-ase, *Drosophila melanogaster *Asense (Genbank: AAF45502); Tc-ase, *Tribolium castaneum *Asense (Genbank: AAQ23387).

We compared the sequences of ASH proteins from silkworm and some other insects, and found that either at whole amino acids level or within the bHLH region, Bm-ASH1 had a higher identity with non-lepidopteran insects' ASH proteins than the other two *Bombyx *proneural proteins, Bm-ASH2 and Bm-ASH3 (Table 1). Among the silkworm ASH proteins, Bm-ASH2 and Bm-ASH3 are most identical, showing 91.3% identity within the bHLH region. It is surprising that Bm-ASH2 and butterfly Achaete-scute homolog1 (B-ASH1) [26] are the same within the bHLH region and share 90.3% identity at the whole amino acid level. Bm-Ase shares more than 70% identities with all of the other insect Asense proteins within the bHLH region. In phylogenetic analysis, proneural proteins and Asense proteins were grouped into two separate clades (Fig. 2). In the proneural clade, Bm-ASH1, Dm-L'sc, Ag-ASH, Am-ASH, and Tc-ASH were grouped into one sub-clade, paralleling the other two sub-clades, one comprised of Dm-Ac and Dm-Sc and the other comprised of Bm-ASH1, Bm-ASH2 and B-ASH1.

### The *Bombyx ASH *genes could not be assembled into a complex based on current data

**Figure 3:**
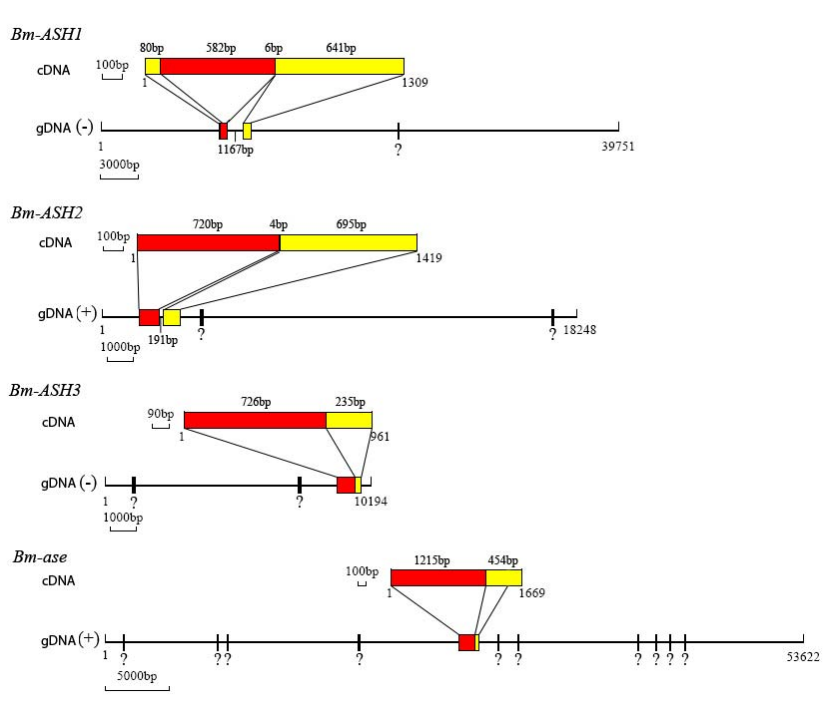
**Genomic structure of *Bm-ASH *genes**. The red region stands for the ORF, and the yellow region stands for non-coding regions; gDNA stands for genomic DNA; (+) and (-) show the published genomic sequences [39] being forward or reverse compared with the cDNAs. "?" shows the region unsequenced. The 6 bp in *Bm-ASH1 *indicates that there is a 6 bp uncoding region in the first exon of the gene just following the coding region, so is the 4 bp in *Bm-ASH2*. Genomic sequences are all from SilkDB [39]. The name of the genomic DNA fragment in *Bm-ASH1 *is Scaffold002070, in *Bm-ASH2 *is Scaffold007910, in *Bm-ASH3 *is Scaffold013050, and in *Bm-ase *is Scaffold000880, respectively.

cDNA sequence of each *Bombyx ASH *gene was used to blast the silkworm knowledgebase [39], and four separate scaffolds were screened out. The total length of the scaffolds is about 122 Kb (but we could not assemble them into one complex with the present data). Analysis results showed that there was an intron outside of the ORF region in *Bm-ASH1 *and *Bm-ASH2*, however there was no intron in either *Bm-ASH3 *or *Bm-ase *(Fig. 3).

### *Bm-ASH *genes regulating promoter activity *via *E-box in Bm-N cells

**Figure 4:**
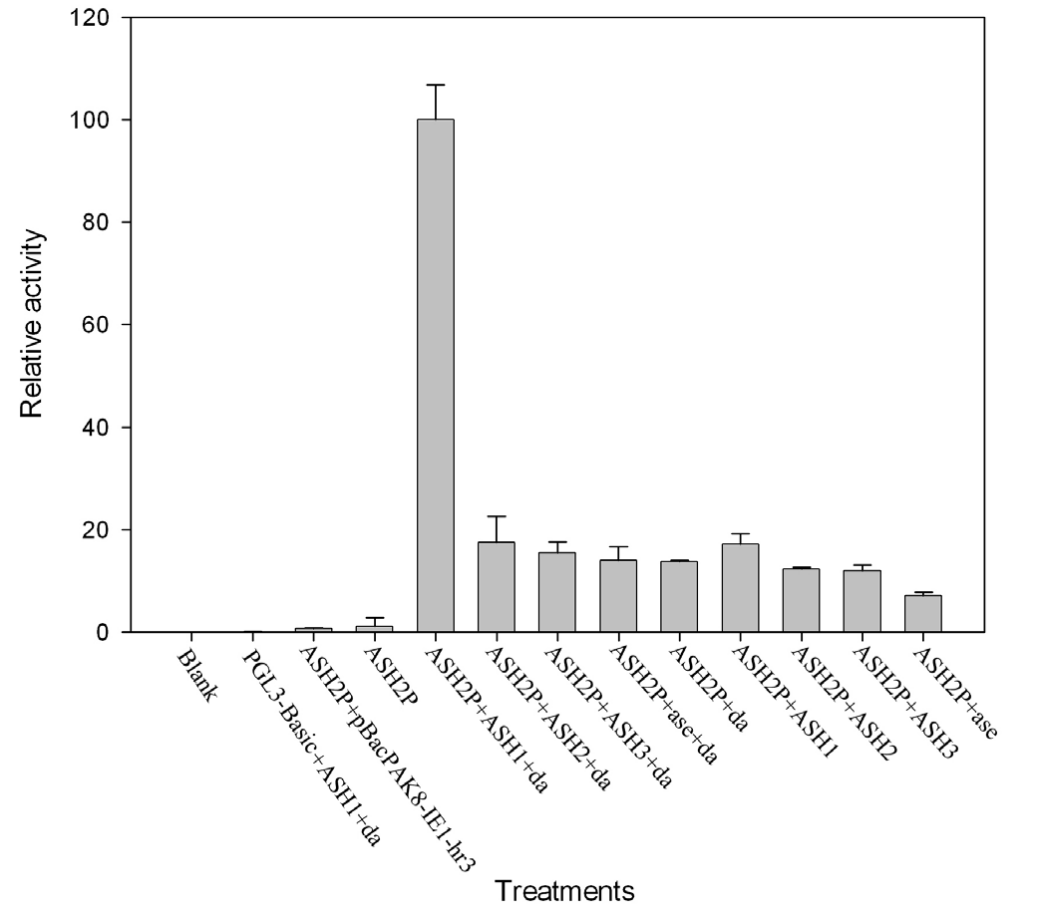
**Activity analysis of ASH2P promoter by co-transfected with *Bm-ASH *genes**. In the figure, ASH2P stands for the recombinanted plasmid pGL3-Basic-ASH2P; ASH1, ASH2, ASH3, ase and da respectively stands for the modified transient expression vector pBacPAK8-ie1-hr3 containing *Bm-ASH1*, *Bm-ASH2*, *Bm-ASH3*, *Bm-ase *and *Dm-da *(*daughterless*) ORFs as transcriptional factor genes. At least three independent repeats were carried out for each treatment.

*AS-C *genes have self-regulating functions by E-boxes in their promoter regions [12]. We cloned a 1,178 bp DNA fragment upstream of the ORF region of the *Bm-ASH2 *gene. At positions 188–194 bp and 791–797 bp upstream of ATG codon, there are two Class A E-boxes (CAGG/CTG). The promoter segments were cloned into pGL3-Basic vector with a luciferase reporter gene, and *Bm-ASH *and *da *genes was cloned into the modified transient expression vector pBacPAK8-ie1-hr3 [40]. Recombined plasmids were co-transfected into Bm-N cells by different assorted ways. We found that the promoter had no activity without the co-transfection of *Bm-ASH *or *da *genes' transient expression vectors (Fig. 4). The promoter had a significantly higher activity by co-transfection with *Bm-ASH1 *and the *da *genes' transient expression vectors than by other co-transfections. Besides, the promoter activity was weaker when it was only co-transfected into cells with *Bm-ASH *genes than when also with *da *(ex., promoter activity of ASH2P+ASH1 was weaker than that of ASH2P+ASH1+da, Fig. 4).

**Figure 5:**
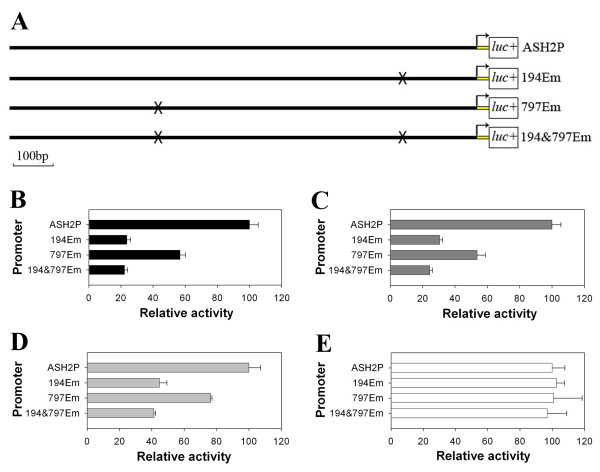
***Bm-ASH *genes enhance ASH2P Promoter activity *via *E-boxes**. ASH2P, the promoter segment of *Bm-ASH2*; 194Em, ASH2P mutated in the E-box 188–194 bp upstream from the start codon; 797Em, ASH2P mutated in the E-box 791–797 bp upstream from the start codon; 194&797Em, ASH2P mutated in both of the E-boxes. Forks show the mutated E-box site (A). B, C, D and E are the results co-transfected of promoters and da with ASH1, ASH2, ASH3 and ase, respectively. E-boxes mutation significantly reduced the promoter activity in B, C and D (*p *< 0.01), but had no effect in E (*p *> 0.05).

In order to study whether the bHLH transcriptional factors regulate the promoter by the E-box region, we changed one or both of the deduced E-boxes to "aAGG/CcG" by site-directed mutation (Fig. 5A). Then the mutated promoters were cloned into pGL3-Basic vector as described above, and co-transfected Bm-N cells with each *Bm-ASH *and *da *genes' transient expression vectors, respectively. The results showed that E-box mutation significantly reduced the activity of the promoter when co-transfected with silkworm proneural genes (Fig. 5B, C, D; *p *< 0.01). However, the activation of *Bm-ase *did not seem to be related with either of the two deduced E-boxes (Fig. 5E; *p *> 0.05). These data suggest that proteins encoded by *Bm-ASH *genes have similar functions to the AS-C transcriptional factors in *Drosophila*, and they synergize with Da protein.

### Expression distribution of *Bombyx ASH *genes in larval tissues

**Figure 6:**
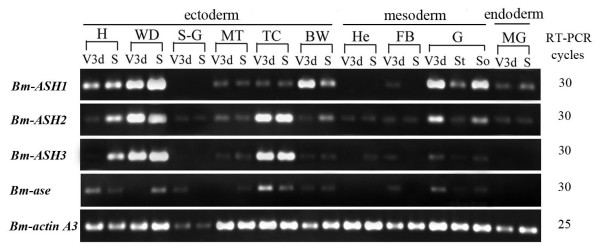
**Tissue expression distribution of *Bm-ASH *genes in silkworm larvae**. RT-PCR templates including: ectoderm tissues H (head), WD (wing disc), S-G (silk gland), MT (Malpighian tubule), TC (trachea cluster), BW (body wall); mesoderm tissues He (hemocyte), FB (fat body), G (gonad, T (testis), O (ovary)); and endoderm tissue MG (midgut). V3d stands for 3-day-old larvae of the 5th instar, and S stands for 8-day-old larvae of the 5th instar (begin spinning). Amplification cycles were 30 for *Bm-ASH1*, *Bm-ASH2*, *Bm-ASH3 *and *Bm-ase*, and 25 for the internal control gene *Bm-actin A3*.

To understand the potential in vivo regulations of *ASH *genes, we detected the expression status of *Bombyx ASH *genes in different tissues of the 5th instar larvae on the 3rd and the 8th days (just after spinning) (Fig. 6). The data showed that *Bm-ASH1 *and *Bm-ASH2 *were expressed in organs derived from all the three cellular derms: ectoderm, mesoderm and endoderm; whereas *Bm-ASH3 and Bm-ase *were only expressed in tissues derived from ectoderm and mesoderm. *Bm-ASH1 *was expressed in all organs except silk glands (S-G) and hemocytes (He), and at a low level in 5 d and had no expression in 8 d fat body (FB). *Bm-ASH2 *was widely expressed in all the investigated samples. The expression of *Bm-ASH3 *and *Bm-ase *were more tissue specific, and the level of *Bm-ase *was relatively lower. *Bm-ASH1 *and *Bm-ASH2 *had high expression levels in gonads (G), while *Bm-ASH2 *and *Bm-ASH3 *had high expression levels in tracheal clusters (Tc). All of the data suggested that the four *Bm-ASH *genes might play various roles in the development of silkworm larva. We should note that all three proneural genes expressed highly in WD, and *Bm-ase *had a relatively higher expression level in 8 d WD. These implied that *Bm-ASH *genes might be important for the development of the wing disc.

### Developmental changes of *Bombyx ASH *genes in the embryo and in the pupal wing

**Figure 7:**
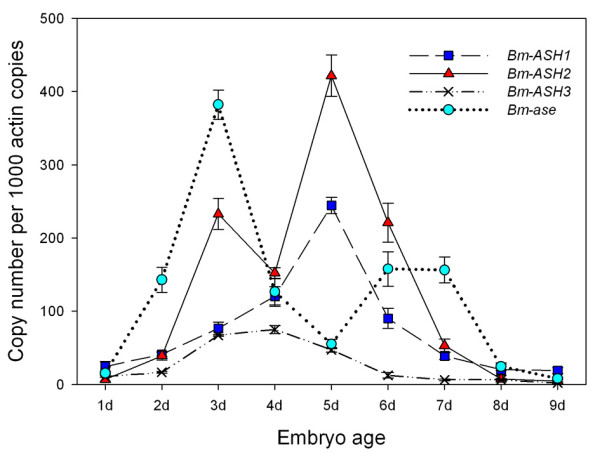
**Developmental changes of *Bm-ASH *genes in silkworm embryo**. The numerical value are copy number of *ASH *cDNA per 10^3 ^*Bm-actin A3 *cDNA copies. At least three independent repeats were carried out for each developmental stage.

Functional genes play their roles by expression in special tissues and at special time. We detected the expression level of *Bombyx ASH *genes in the embryo and pupal wing in order to study the function of these genes during silkworm development. Total RNA was extracted from 1 d to 9 d embryos or from wings/wing discs (from spinning to the adult phase), and used for reverse transcription. cDNA synthesized from about 10 ng total RNA was taken as the template for each PCR reaction. We calculated the gene copy number corresponding to 10^3 ^*Bm-actin A3 *copies at each developmental point in the embryo and 10^6 ^in the wing/wing disc. The results showed that during the embryo development, both *Bm-ASH2 *and *Bm-ase *had two expression peaks, one being both on 3 d, and the other being on 5 d for *Bm-ASH2 *and on 6 d for *Bm-ase *(Fig. 7). However, there was only one peak for each of the other two *Bm-ASH *genes, *Bm-ASH1 *on 5 d and *Bm-ASH3 *on 4 d. Although the expression levels of *Bm-ASH3 *showed a peak from 3 d to 5 d, its expression level was always lower than the other three genes.

**Figure 8:**
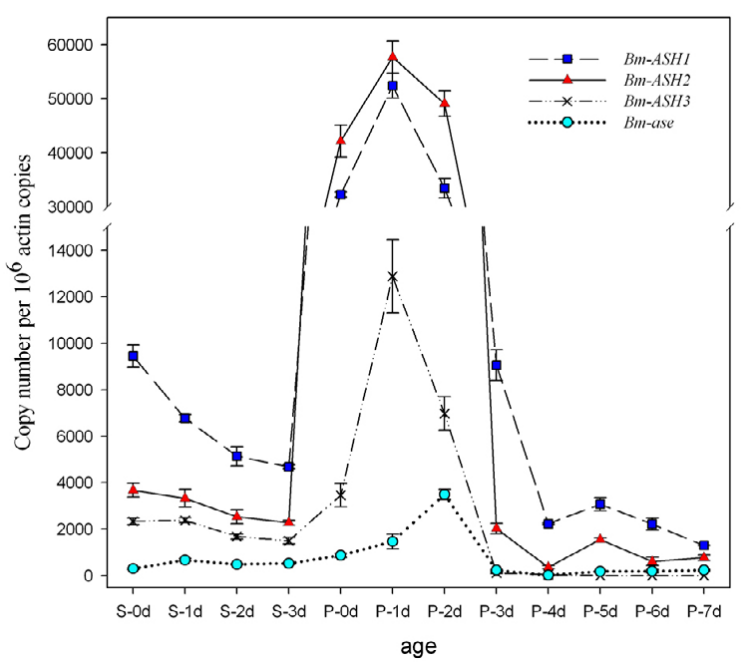
**Developmental changes of *Bm-ASH *genes in silkworm wing disc/wing**. The numerical value are copy number of *ASH *cDNA per 10^6 ^*Bm-actin A3 *cDNA copies. S0-S3 represent days after the beginning of spinning, and P0-P7 represent days after pupation. At least three independent repeats were carried out for each developmental stage.

During the spinning (wandering) and pupal ages, the silkworm wing disc/wing form changes obviously by extension, turning and scale foundation. The results of quantitative RT-PCR showed that all four genes had relatively high expression levels from 0 d to 3 d pupae (Fig. 8). The result of *Bm-ASH1 *was similar to our previous results [41]. *Bm-ASH1*, *Bm-ASH2 *and *Bm-ASH3 *all reached their peak values on P-1 d, whereas *Bm-ase *peaked one day later. Flanking these days, expression levels of the genes were significantly lower. During the spinning period, expressions of all four genes were at low levels, with *Bm-ase *being undetectable. It was notable that the expression levels of the three proneural genes decreased from S-0d to S-3d. *Bm-ASH3 *and *Bm-ase *were not express anymore after P-3d pupa, while both *Bm-ASH1 *and *Bm-ASH2 *had a low but noticeable peak in 5 d pupa.

## Discussion

Our results showed that there were four *AS-C *homologs in *Bombyx mori*: *Bm-ASH1*, *Bm-ASH2*, *Bm-ASH3 *and *Bm-ase*. The first three were proneural genes, and the last one was a neural precursor gene. The proneural genes enhanced the activity of the *Bm-ASH2 *promoter by binding its E-boxes in Bm-N cells, which was one of the *AS-C *homolog characteristics (Figs. 4 &5). The four genes had various expression profiles in silkworm larval tissues (Fig. 6), and further studies showed that they have important roles during the development of the embryo and the wing (Fig. 7 &8).

### *AS-C *genes duplication in insects occurs after or parallel but not before the taxonomic order formation during evolution

Homologs of *AS-C *genes exist in various animals from low-grade coelenterate to high-grade mammals, including human being [30]. It has been proposed that *AS-C *gene has several independent duplication events in Arthropoda, resulting in the plasticity of the gene number. In this model, *Cn-ASH*, the *AS-C *homolog in Hydra is supposed as the ancestral one. Parallel but independent duplication events occurred in insects and chelicerates (Fig. 2,a &2b). Within the Diptera two more duplication events happened during evolution. So the present most derived dipteran, *Drosophila melanogaster*, has the four *AS-C *genes *achaete*, *scute*, *lethal of scute *and *asense *[16,30]; while lower derived dipteran, such mosquito, has only two [31].

We found that there were four *AS-C *homologs in the silkworm, *Bombyx mori*, a model lepidopteran. Also, *Bombyx *is the only insect besides *Drosophila *found with four *AS-C *genes so far. Among the three proneural *AS-C *genes in *Drosophila*, *l'sc *is the most original one, which is more identical to the proneural genes in other insects than *ac *and *sc *(Table 2). Like *l'sc*, *Bm-ASH1 *is more identical to non-lepidopteran insect proneural genes than other silkworm *achaete-scute *homologs (Table 2). What's more, *l'sc *and *Bm-ASH1*, and other sole proneural genes from other insects were sorted into one clade in the phylogenetic tree (Fig. 2). The data imply that *Bm-ASH1 *is the most original one in *Bombyx*. But homogeneous and phylogenetic analysis could not match each *Bombyx *proneural gene to those of *Drosophila *one by one, and phylogenetic result sorted *ac/sc *and *Bm-ASH2/Bm-ASH3 *into two independent branches. So we suggest that the generations of *ac/sc *from *l'sc *and *Bm-ASH2/Bm-ASH3 *from *Bm-ASH1 *are two parallel and independent events, that is, further duplication of proneural genes occurs after or parallel to but not before the split of Diptera and Lepidoptera during evolution.

**Table 2:** Homology comparison of proteins encoded by the *Drosophila AS-C *genes with some of the other insect ASH proteins.

**ASH name**	**Percent Identity (%)**		
	
	**Dm-L'sc**	**Dm-Sc**	**Dm-Ac**
**Dm-L'sc**	/		
**Dm-Sc**	73.5 (34.6)	/	
**Dm-Ac**	69.1 (35.8)	66.7 (33.8)	/
**Ag-ASH**	79.4 (43.2)	73.5 (29.0)	75.0 (37.3)
**Am-ASH**	72.1 (26.6)	69.6 (28.9)	66.7 (33.3)
**B-ASH1**	73.5 (31.9)	62.3 (25.2)	66.7 (29.9)
**Bm-ASH1**	74.6 (40.9)	68.7 (42.0)	68.7 (31.6)
**Bm-ASH2**	72.1 (31.0)	62.3 (27.2)	65.7 (28.9)
**Bm-ASH3**	72.1 (34.9)	58.0 (27.4)	65.2 (30.3)
**Tc-ASH**	70.6 (34.2)	65.2 (33.2)	63.9 (33.8)

The number in the bracket is the identity percent between two whole protein sequences, and the number before the bracket is the identity percent within the bHLH regions.

The medfly *Ceratitis capitata*, a member of family Tephritidae, has three *AS-C *homolog genes *Cc-sc*, *Cc-l'sc *and *Cc-ase*. The bHLH domains of proteins coded by *Ceratitis capitata acheate-scute *homologs are highly conserved and display 95%, 91.5% and 90% identity with the *Drosophila *counterparts, respectively [28]. Only one *AS-C *homolog, *B-ASH1*, has been isolated from the Nymphalidae insect *P. coenia *[26]. B-ASH1 protein has a surprising identity with Bm-ASH2, 100% within the bHLH domains and 90.3% within the whole protein sequences. It is even higher than that between Bm-ASH2 and Bm-ASH3, 91.3% within the bHLH domains and 49.8% within the whole protein sequences (Table 1). Moths first appeared on earth between 100 and 190 million years ago, and butterflies appeared about 40 million years ago, based on fossilized evidence. All above suggest that there are at least three *AS-C *genes in *P. coenia*, corresponding with *Bm-ASH1*, *Bm-ASH2 *and *Bm-ase*, respectively, and *Bm-ASH3 *might be the most recent one among the silkworm proneural genes.

### *AS-C *genes have a broad expression distribution in insect tissues

*AS-C *genes are important for the development of the nervous system, and have key roles in regulating the formation and patterning of neural precursors. They are specially expressed in most of the proneural clusters during the development of either the central (CNS) or peripheral nervous system (PNS) in arthropod, such as flies, butterflies, beetles, bees, spiders, chilopods, etc. Other functions of these genes have also been found in *Drosophila*. *l'sc *participates in the specification of muscle progenitors [21], *sc *functions in sex determination [18] and *ac *regulates the development of Malphigian tubules [17].

In the present paper, we studied the expression of *Bm-ASH *genes in various silkworm larval tissues using RT-PCR methods. These genes are expressed in most organs derived from all three derms, showing they might have multiple functions during silkworm development. They have higher expression levels in the head, wing disc, tracheal cluster, body wall or gonad, and all of them are expressed in the organs though lower at some developmental points. This indicates that the genes might have overlapping functions, just as in *Drosophila *[42]. Expression profiles during the development of the embryo and the pupal wing also suggest co-operation characters of *Bm-ASH *genes. During silkworm embryo development, neurogenesis takes place around 3 d-old, and trachea, bristle and appendage occur on 5 d and 6 d. The correspondence between the two stages and those when the two expression peaks of *Bm-ASH *genes occure in the embryo (Fig. 7) suggests that these genes regulate the development of PNS and CNS. Expressions in wings of all the four genes are significantly higher on 1 d or 2 d after pupation than during other stages. Wing scale precursor cells form around the 2 d-old pupal stage [41], and our further studies on the scaleless wings mutant silkworm strongly proved the key role of *Bm-ASH2 *in the formation of wing scales (Zhou et al., unpublished).

Although we have known many important functions of *AS-C*, the analysis of the structures and evolution of these genes may suggest some of their unknown functions. We compared the protein sequences of 38 *AS-C *homologs from vertebrate and invertebrate. There are conserved domains besides the bHLH and C-terminal motifs, especially within vertebrates. This conservation even exists between vertebrate and invertebrate organisms. A ~20 amino acid conserved domain (corresponding to PEMRCKRRINFAQLGYNLPQ of Asp-ASH) was found in *ASH *genes from vertebrates and myriapod animals *Lithobius forficatus *(*Lf-ASH*, Genbank: AAT99570) and *Archispirostreptus *(*Asp-ASH*, Genbank: AJ536345). Together with the broad expression of *Bm-ASH *genes in tissues outside of the nervous system, they show that this gene family has other important functions waiting for exploration.

## Conclusion

In this work, we isolate and identify four *achaete-scute *homologs from *Bombyx mori*. So far, *Bombyx mori *is the second insect which has been found to have four *AS-C *genes. During organism evolution, genes are duplicated with conserved domains to gain more special functions. Results of phylogenetic and gene expression analysis show that during evolution, *AS-C *genes duplication in insects occurs after or parallel to but not before the taxonomic order formation and functions of these genes are broad during insect development.

## Methods

### Insects and cell culture

The silkworm stock Jingsong × Haoyue was maintained in our laboratory. The insects were reared on an artificial diet at 25°C with 70%–80% relative humidity. Bm-N cells derived from silkworm were cultured at 27°C in TC-100 insect medium containing 10% heat-inactivated (56°C, 30 min) fetal bovine serum (Invitrogen). Cell culture details were the same as Summers and Smith [43].

### RNA isolation and RT-PCR

Silkworm tissues were dissected out at different stages and the total RNA was extracted with TRIZOL Reagent (Invitrogen) according to the standard protocol, and whole embryo total RNA was extracted using the acid-guanidine method [44]. One microgramme of total RNA from each sample was used to synthesize first-strand cDNA using M-MLV Reverse Transcriptase (Promega) as the protocol described. PCR with proper program was performed using the reverse transcription product as template. Sequences of all primers used in this paper are available upon request.

### Rapid amplification of cDNA ends (RACE)

One microgramme of total RNA was used for RACE cDNA synthesis (BD SMART™ RACE cDNA Amplification Kit, Clontech), according to the user's manual. PCR was performed with primer1 and Universal Primer A Mix (UPM, Clontech), then a nest PCR was processed with primer2 and NUP using the suitable diluted former PCR product as the template. Each PCR reaction was carried out under the following conditions: one cycle of pre-denaturing for 5 min at 95°C; and 30 cycles of 94°C for 40 s, 60°C for 40 s, 72°C for 3 min, then followed by 10 min incubation at 72°C.

### Database blast, protein sequences alignment and phylogenetic analysis

We used the amino acid sequence coded by *Drosophila achaete *(Genbank: AAF45498) to blast the silkworm EST database with the blastx program on the NCBI web site [45], by limiting the organism with "*Bombyx mori*". As formerly described [46], insect *ASH *protein sequences were aligned with CLUSTALX [47] and revised manually with Gendoc software. Then a neighbor-joining (NJ) tree based on amino acid sequences was constructed using the PHYLIP software package (100 bootstrap replicates) [48].

### Dual-Luciferase Reporter Assay

Promoter segments were cloned into Luciferase Reporter Vector pGL3-Basic separately, and the ORF region of silkworm *ASH *and *Drosophila da *(GenBank: Y00221) genes were cloned into the modified pBacPAK8 vector with an *IE1 *promoter and a *hr3 *enhancer [40]. Then the plasmids were transfected into Bm-N cells as described formerly [49]. 0.1 μg of pRL-CMV Vector was co-transfected as an internal control reporter for each transfection. After incubating for 48 hours, the cells were collected by centrifugation at 10,000 rpm for 1 min at 4°C. Then the cell lysates were prepared using the passive lysis buffer, and 10 μg of each lysate was used for the dual-luciferase reporter assay according to the protocol (Dual-Luciferase^® ^Reporter [DLR™] Assay System, Promega). Firefly luciferase activity and Renilla luciferase activity were determined with 20/20^n ^Luminometer (Turner BioSystems, Inc., USA). Each treatment was repeated at least three times.

### Introduction of point mutation

Primers P-F and P-R were designed at the terminals of the target segment sequence. Reversed primers m-R and m-F with the mutated bases were designed around the site where the point mutation would be introduced. PCRs were carried out with P-F pairing m-R or m-F paring P-R. After purification, the two PCR products were mixed, and denatured, renatured and extended for three cycles without any primers. Three cycles after running, P-F and P-R were added and the PCR was continued for 30 cycles of amplification. All of the PCRs were processed with *pfu *DNA polymerase.

### Quantitative real-time PCR

Q-PCR was used to determine the changes of silkworm *ASH *genes expression during embryo development and wing development. Primers were designed based on the cDNA sequence and a segment around 200 bp would be specially amplified for each gene. The housekeeping gene *Bm-actin A3 *was used as the internal control. A 20 μl volume containing cDNA produced from about 10 ng of total RNA, 5 pmol of each primer, and 10 μl of SYBR Green Realtime PCR Master Mix (Toyobo Co., Ltd., Japan) was used for each PCR reaction. Then the PCR was processed on a Chromo4 Four-Color Real-Time System (Bio-Rad [formerly MJ Research]) under the following program: one cycle of 95°C for 3 min; then 40 cycles of 95°C for 15 sec, 60°C for 15 sec and 72°C for 30 sec. The melting curve was established from 60°C to 95°C. Three independent repeats were carried out for each reaction. Threshold cycle values were used for the further analysis.

**Figure 9:**
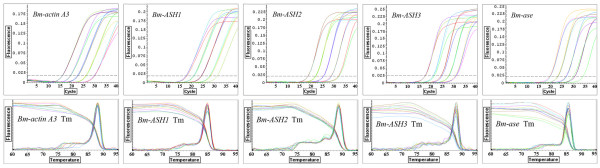
**The data curves (upper) and melting curves (lower) of Quantitative real-time PCR for *Bm-actin A3 *and *Bm-ASH *genes**. These are the curves of amplifications for standard curve making. The plots show that the repeatability and specification of the Q-PCR are good.

**Figure 10:**
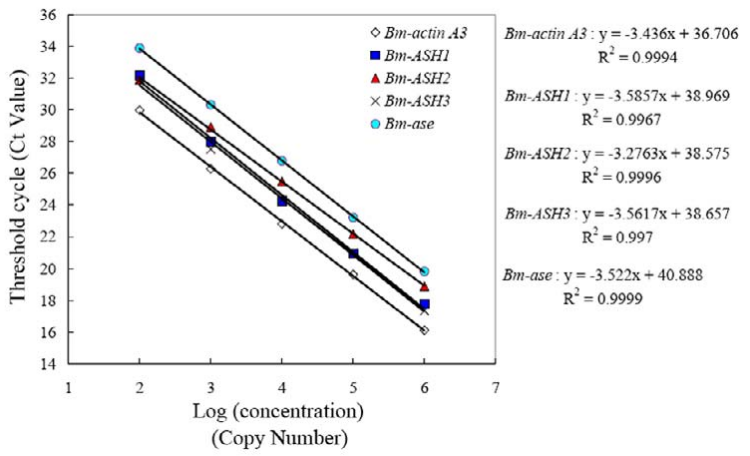
**Standard curves of the Quantitative real-time PCR**. Standard curves were constructed using serial dilutions of the ORF segments of the genes recombined into pMD18-T vectors. The log ranges of the standard curves are from 10^6 ^to 10^2 ^copies. Threshold cycles of each standard dilution point were used to generate the standard curve for each gene. Linear equations corresponding to each standard curve are given, and R^2 ^for each standard curve is more than 0.99.

Standard curves were constructed using serial dilutions of the ORF segments of the genes recombined into pMD18-T vectors (Takara, Japan). The log ranges of the standard curves were from 10^6 ^to 10^2 ^copies. The data curve and the melting curve showed the reliability and specificity of the PCR product (Fig. 9). Threshold cycles of each standard dilution point were used to generate a reliable standard curve for each gene (Fig. 10). Linear equations corresponding to each standard curve were made, and R^2 ^for each standard curve was more than 0.99. This showed that the linearity of the standard curves was good.

Theoretic copy of each sample was calculated with the linear equation for each gene, and trendlines for *Bombyx ASH *genes during the development course of silkworm embryo and pupal wing were constructed.

## Authors' contributions

ZQX carried out most of the work and wrote the paper. ZTY isolated *Bm-ASH1 *and helped write the paper. YLL took part in RT-PCR and helped analyzing the data. YYZ cultured the cells. XWH and ZZF conceived the project and draft the manuscript. All authors read and approved the final manuscript.
